# Knowledge and attitudes about epilepsy of neurology outpatients in Türkiye

**DOI:** 10.55730/1300-0144.6068

**Published:** 2025-08-19

**Authors:** Ayşe Pınar TİTİZ, Abidin ERDAL, Yaren AĞAR, Buse ALTUNBAŞAK

**Affiliations:** Department of Neurology, University of Health Sciences, Ankara Bilkent City Hospital, Ankara, Turkiye

**Keywords:** Epilepsy, knowledge, attitude, stigma, awareness

## Abstract

**Background/aim:**

We aimed to evaluate the level of epilepsy-related knowledge and attitude among neurology outpatients without an epilepsy diagnosis.

**Materials and methods:**

The Epilepsy Knowledge and Attitude Scale was applied to 331 adult patients who were evaluated with various diagnoses in the neurology outpatient clinic.

**Results:**

The participants comprised 331 people, 51.4% of whom were women and 48.6% of whom were men. The knowledge and attitude values increase significantly as the education level of our participants increased (p < 0.001). The knowledge and attitude scores of those who knew about an individual with epilepsy were significantly higher (p < 0.001). Attitude scale scores were higher in patients diagnosed with headache, multiple sclerosis, syncope, Behçet’s disease, essential tremor and sleep disorders compared with other groups.

**Conclusion:**

This study focused on a subgroup of adults without epilepsy who had other neurological conditions. The findings may illuminate the critical role of education and public awareness in fostering more positive attitudes toward individuals with epilepsy, reducing stigma, and facilitating their social integration.

## 1. Introduction

Epilepsy ranks among the most widespread neurological disorders both on a global scale and within our nation. It is a complex disease with a paroxysmal course in which various symptoms occur during the seizure period. It may occur across all age groups and affects an estimated 50 million people in the world. The incidence of epilepsy is approximately 50–60 cases per 100,000 person-years. At least 8% of people experience one seizure during their lifetime [1]. Seizures are controlled in approximately two-thirds of patients receiving appropriate and regular anti-seizure treatments [2]. Although epilepsy can be highly disabling when seizures are frequent, effective seizure control is achievable through appropriate medical management and good compliance of the patient with treatment. Nonetheless, if patients and the individuals they interact with in society hold misconceptions or negative attitudes regarding epilepsy, their social participation and quality of life may be adversely affected. In addition to appropriate drug treatment for epileptic individuals, a high standard of social well-being can also contribute positively to the control of seizures [3].

Many patients with epilepsy may be reluctant to disclose their condition because of the stigma, exclusion, and social anxiety it may cause. As epileptic individuals are exposed to self-stigma and stigma from their surrounding environment, “epilepsy awareness” has been the subject of many studies to date, and the management of knowledge, attitudes and behaviors in society regarding this issue has become increasingly important over time [4]. Given that epilepsy is a lifelong condition, it is crucial for the relatives of patients, as well as the individuals they interact with in society, to possess adequate awareness and understanding of the disease [5]. Studies assessing the knowledge and attitudes toward epilepsy across various segments of society further substantiate this finding. These studies have revealed varying results across different societies and cultures [3, 4, 5].

Previous studies conducted in Türkiye on this subject have predominantly focused on healthcare professionals or family members residing with epilepsy patients. In this study, we aimed to comparatively evaluate the knowledge and attitudes of neurology patients without an epilepsy diagnosis regarding epilepsy, according to age, sex, education level, and the presence of a relative with epilepsy.

## 2. Materials and methods

### 2.1. Study design, participants

This cross-sectional study included 331 adult patients without an epilepsy diagnosis who met the inclusion criteria and voluntarily completed the questionnaire via face-to-face interviews at the Neurology Outpatient Clinic of Ankara Bilkent City Hospital between December 2023 and February 2024.

Individuals who had been previously diagnosed with epilepsy or had experienced at least one epileptic seizure in their lifetime were excluded from the study. There were several methodological and conceptual reasons for selecting nonepileptic neurology outpatients as the study population: Including patients with other neurological diagnoses allowed for the recruitment of a sample representing a randomly selected subgroup of the adults without epilepsy.

In recent years, increased access to social media platforms and artificial intelligence–based information tools has increased the frequency with which individuals with medical conditions seek health-related information through these sources. Patients with neurological conditions other than epilepsy, may encounter information about epilepsy and seizures while seeking details about their own disorders, particularly when their diagnoses overlap with or resemble epilepsy.

Consequently, individuals in this group may differ from the general population in terms of their knowledge and attitude levels toward epilepsy. Importantly, individuals with neurological conditions other than epilepsy often face similar societal challenges, such as stigma, misunderstanding, and marginalization. This shared sensitivity can foster empathy for others with neurological conditions, including epilepsy, and lead to more thoughtful or compassionate attitudes. Researching this group can offer insights into socially informed societal perceptions and how neurological patients relate to each other’s experiences.

This nonepileptic group also serves as a useful baseline for future comparative studies, allowing for meaningful cross-group analyses of attitudes, awareness, and stigma.

Patients diagnosed with epilepsy were excluded from this study to emphasize a more objective assessment of knowledge and attitudes towards epilepsy.

Aydemir’s, “The Epilepsy Knowledge and Attitude Scale” was applied to each participant to evaluate their epilepsy knowledge and attitude through face-to-face evaluations [6]. The questionnaire consisted of three parts. In the first part, participants were asked about their age, sex, marital status, educational and economic status, prior knowledge of the condition, and if they knew anyone diagnosed with epilepsy; all responses were recorded. The second part of the questionnaire included the epilepsy knowledge scale, and the third part featured the epilepsy attitude scale.

### 2.2. Measurement tools of knowledge and attitude scales

#### 2.2.1. Epilepsy knowledge scale

The epilepsy knowledge scale consisted of 16 items to assess knowledge about epilepsy, as shown in Table 1. Some of these items contain true information, while others contain false information. To evaluate these items, participants were given the options of “true”, “false”, or “I do not know”. Participants with appropriate responses were evaluated and recorded, with each item receiving a score of 1. The total score ranges from 0 to 16, with higher scores indicating greater knowledge about epilepsy.

#### 2.2.2. Epilepsy attitude scale

The epilepsy attitude scale consisted of 14 items and is presented in Table 2. Participants were given the following options: “strongly agree”, “agree”, “no opinion”, “disagree” and “strongly disagree”. Based on their chosen options, the appropriate scores corresponding to participants’ responses were recorded on a scale of 1 to 5, with 1 representing the worst attitude and 5 representing the best attitude. The total score on the epilepsy attitude scale was 70, assuming the best possible attitude.

The “total knowledge” and “total attitude” values were collected and recorded according to the knowledge and attitude evaluations of each individual to whom the survey was applied. Ethics committee approval was obtained from Ankara Bilkent City Hospital Clinical Research Ethics Committee No. 2 on November 22, 2023 (Approval No. E2-23-5685).

### 2.3. Statistical analyses

Data were analyzed using IBM SPSS V23. The suitability of the data for normal distribution was examined using the Kolmogorov-Smirnov test. The Mann-Whitney U test was used to compare data that did not show a normal distribution according to binary groups. Analysis results are presented as frequency (percentage) for categorical variables, and mean ± standard deviation and median (minimum –maximum) for quantitative variables. The significance level was set at p < 0.050. Spearman statistical method was used for nonparametric correlations. The evaluation of knowledge and attitude levels between the diagnosis groups was performed using a one-way ANOVA statistical test.

## 3. Results

The participants consisted of 331 people, 170 (51.4%) of whom were women and 161 (48.6%) were men. Their ages ranged from 18 to 68. Considering the participants’ diagnoses, the highest rate was headache (30.8%), followed by cervical radiculopathy (15.1%), and vertigo (13.9%). When we looked at marital status distribution, the highest rate was for those who were married (54.7%). Considering the highest level of education attained, the largest proportion of participants were high school graduates (45.9%). For analytical purposes, participants were categorized into two groups based on their education level: those with lower education (defined as high school or below), comprising 51.7% of the sample, and those with higher education (defined as university degree or above), comprising 48.3%. When looking at the distribution of employment status, the highest response “I am working”, reported by 61% of participants. Meanwhile, 53.2% of participants were under the age of 40, while 46.8% were aged 40 and over was 46.8%, as shown in Table 3. The mean age of the patients was 38.93.

The proportion of participants who had witnessed an epileptic seizure was 48.3%, and 74% of people had heard of epilepsy prior to the study. The rate of people who heard about epilepsy from the internet was 36.6%, whereas 63.4% had learned about it from people around them. The proportion of those who had read something about epilepsy before was 39.6% and 82.4% of individuals had read about epilepsy on the internet. Some participants had read about epilepsy in a book, accounting for 17.6% of the sample. The percentage of people who knew of someone with epilepsy was 54.1%. In this group, 38% had a friend with epilepsy, 21.8% had relatives with epilepsy, 8.9% had family members with epilepsy, 17.3% had neighbors with epilepsy, and 14% knew someone in a hospital setting, as shown in Table 4.

The average total score on the knowledge scale was 9.64, whereas the average total score on the attitude scale was 60.54. There was no statistically significant difference between the knowledge scale total score median values according to the age group (p = 0.377). The median total knowledge scale score was 10 for participants both under 40 years and those aged 40 years or older. There was a statistically significant difference between the knowledge scale total score median values according to the education level (p < 0.001). While the median total score of the knowledge scale for those with a low education level was 9, it was 11.5 for those with a higher education level. There was a statistically significant difference between the knowledge scale total score median values according to knowledge of someone with epilepsy (p < 0.001). While the median total score on the knowledge scale for those who did not know anyone with epilepsy was 9, it was 11 for those who knew someone with epilepsy. There was no statistically significant difference between the knowledge scale total score median values according to sex (p = 0.638). The median total score of the knowledge scale was 10 for women 10 and for men as shown in Table 5.

There was no statistically significant difference between the median values of the attitude scale total score according to the age group (p = 0.053). The median total attitude scale score was 62.5 for participants under 40 years and 62 for those aged 40 or older.

There was a statistically significant difference between the total score distributions of the attitude scale according to education level (p = 0.046). Attitude scores were better in those with a higher level of education. The median total score on the attitude scale for both the low and high education levels was 62. There was a statistically significant difference between the attitude scale’s total score median values according to knowing someone with epilepsy (p < 0.001). Attitude scores were found to be better in those who know someone with epilepsy than in those who do not. While the median total score on the attitude scale of those who did not know anyone with epilepsy was 61, it was 63 for those who knew someone with epilepsy. There was no statistically significant difference between the attitude scale total score median values according to sex (p = 0.947). The median total score on the attitude scale for both men and women was 62 as shown in Table 6.

It was observed that as the total score on the patients’ knowledge scale increased, their total scores on the attitude scales increased. A moderately positive relationship was observed for the correlation coefficient (r = 0.429).

**Figure 1:**
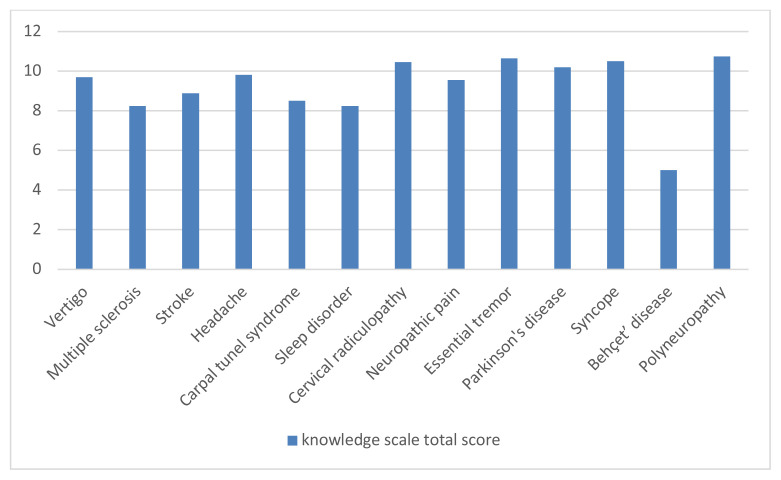
Assessment of attitudes among the different diagnostic groups.

No statistically significant differences were found between the diagnostic groups in terms of epilepsy knowledge and attitude scores. However, relatively higher attitude scores were observed in patients with diagnoses such as headache, multiple sclerosis, syncope, Behçet’s disease, essential tremor, and sleep disorders as shown in Figure 1. While these differences were not statistically significant, they may reflect varying levels of familiarity with neurological conditions or prior exposure to information about epilepsy during medical evaluations. For example, patients with syncope are often investigated to rule out epileptic seizures, which may lead to increased awareness through the diagnostic process. Conversely, lower knowledge and attitude scores were noted in stroke patients (n = 19), a group with a relatively high risk of developing epilepsy. Although this risk is well-documented in the literature, it is not always emphasized in clinical practice, and stroke patients may not consistently receive information about it. Additionally, the small sample size in this subgroup may have limited variability and reduced the ability to detect meaningful differences.

In the knowledge scale, the most accurate response was to the statement: “Most children with epilepsy can go to public schools” (78.9%), followed by “Inadequate sleep, stress, and drinking alcohol can cause a seizure” (78.5%) and “Patients with epilepsy can be dangerous to others during a seizure” (74.3%) as shown in Table 1.

The most inaccurate response was to the statement “Some kinds of seizures can hard to notice by others” (75.2%), followed by “There are many different types of epilepsy” (74.3%) and “Some seizures may last for a matter of seconds” (60.1%).

For the attitude scale, responses of “strongly agree” or “agree” to favorable items were classified as positive attitudes, while “disagree” or “strongly disagree” responses were classified as negative attitudes. The reverse was done for unfavorable attitudes. Accordingly; in the attitude scale evaluation, the most positive response was for the statement “Having epilepsy is something to be embarrassed about” (95.2%), followed by “I would stay away from a friend if I know she/he had epilepsy” and “I feel uncomfortable working with someone who has epilepsy” (both 94.6%). The most negative response was for the statement “I would date someone who has epilepsy” (18.2%), followed by “I would marry someone who has epilepsy” (15.7%) and “If I had epilepsy, I would hide it from my friends” (13.6%) as shown in Table 2.

## 4. Discussion

Epilepsy-related knowledge and attitudes were evaluated in 331 participants (170 women, 161 men) with neurological disorders other than epilepsy. When analyzing the diagnoses given to the participants, the highest rate was headache with 30.8%. In terms of marital status distribution, the highest rate was found among married individuals, at 54.7%. In the study by Aydemir, which served as the source for the scale used in our research; the distribution of marital status was similar to that in our study, with the highest proportion of participants being married individuals, reported at 53.9% [6]. The rate of individuals who had heard of epilepsy before was 74.9%, whereas 39.6% had read something about epilepsy previously. In similar studies conducted in Türkiye, 68.4% had heard or read about epilepsy, 44% knew something about epilepsy [7], 58.8% had heard about epilepsy, and 26.3% had read sources about epilepsy [8]. In another study conducted in Türkiye involving 1354 participants, three-quarters had heard of epilepsy, and half reported knowing someone with the condition. [6]. In another study conducted in Thailand involving 1581 participants, 80.8% were familiar with the word “epilepsy”, but only a small proportion personally knew someone with this condition [9]. Similarly, in a study of 456 participants in Cameroon, 72.6% of participants had heard of epilepsy or knew someone with epilepsy, 76.8% reported having had seizures, but 13% still described the condition as a “state of insanity” due to lack of knowledge [8]. While our patients mostly heard about epilepsy from their surroundings (63.4%), the most common source was the internet (82.4%). In similar studies conducted in Türkiye, the most common source was the internet [8, 10]. While the knowledge scale in our study did not show significant differences between the younger and older age groups, another study highlighted that young individuals tend to have a better level of knowledge compared to the elderly population [10]. Similar to Kurt’s study, our research found no statistically significant difference in the total attitude scale scores across age groups [8]. However, another study conducted in Türkiye highlighted that younger age groups tend to have more positive attitudes [6]. Although our study found no significant differences in knowledge and attitude scores based on sex, several studies have highlighted a correlation between the male sex and higher attitude levels [6, 11, 12]. Consistent with previous studies, knowledge and attitude scores increased significantly with higher educational attainment (p < 0.001) [8,10,12]. The knowledge and attitude scores of those who knew about an individual with epilepsy were significantly higher (p < 0.001). The findings in the literature are in agreement with these results [9, 12].

When the relationship between the total scores of the participants’ knowledge and attitude scales was evaluated, a positive correlation was observed (r = 0.429). The results of other studies evaluating epilepsy knowledge and attitudes are similar [6, 8, 9, 12]. A recent study conducted in Türkiye emphasized that individuals with higher levels of education, particularly those with a background in health education, demonstrated greater knowledge and more positive attitudes toward epilepsy, as expected [13].

In the attitude scale evaluation, the most negative response was to the statement “I would date someone with epilepsy” (18.2%), followed by “I would marry someone with epilepsy” (15.7%). A review of the relevant literature reveals contrasting findings. A study published in 2015 evaluating attitudes toward individuals with epilepsy reported that participants did not perceive significant barriers related to employment or marriage [14]. In contrast, a study conducted in Cameroon found that 39.6% of participants did not want their children to associate with someone who has epilepsy, and 33.6% were opposed to the idea of their children marrying individuals with epilepsy [8]. Similarly, another study conducted in our country also revealed that participants held negative attitudes toward marriage with individuals with epilepsy [7]. Unfortunately, these results support the justified concerns of individuals with epilepsy regarding stigma and sociocultural exclusion.

When we look at the attitude scale evaluation of 16 participants with epilepsy in their families, the most negative response was to the statement, “If I had epilepsy, I would hide it from my friends”, followed by “I would marry someone with epilepsy” and “I would object to my child marrying someone with epilepsy.” These results indicate that stigma related to epilepsy persists even within the family environment.

Although there was no statistically significant difference between the diagnostic groups in our study in terms of epilepsy knowledge evaluations, it was observed that the attitude scale results were better in the groups with headache, multiple sclerosis, syncope, Behçet’s disease, essential tremor, and sleep disorders than in the other groups. Since no comparable studies have made this specific evaluation it is not currently possible to interpret the findings within the context of existing literature. However, it may be inferred that this group’s level of empathy could be higher, considering that individuals with such diagnoses similar to epilepsy patients are predominantly young, experience paroxysmal symptoms, and remain completely healthy between episodes. Further studies are needed to validate and expand upon this interpretation.

**Figure 2:**
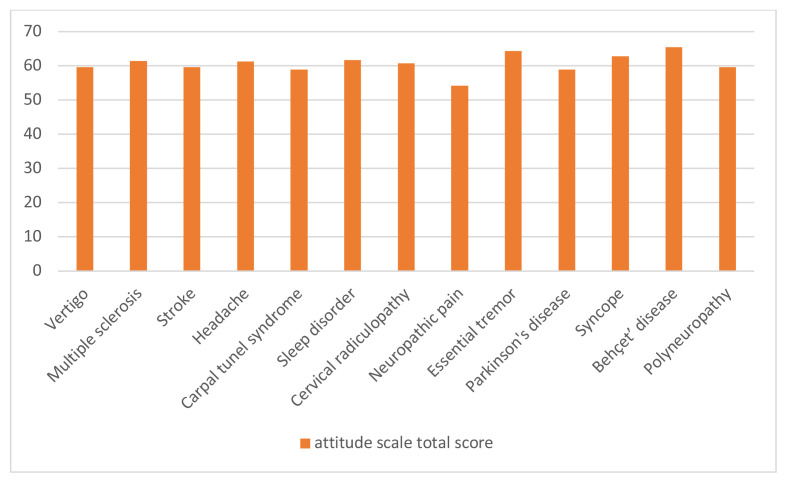
Assessment of knowledge among the different diagnostic groups.

In our study, we reviewed the patients’ data based on their diagnoses. Subsequently, we emphasized the data of patients whose diagnoses exhibited a higher prevalence of coexistence with epilepsy. The patients, who constituted the largest group in our study (30.8%), were diagnosed with headache. While there was no notable disparity between the diagnosis groups, the attitude and knowledge scale scores were particularly high in the headache group, as illustrated in Figure 1 and Figure 2. Recent epidemiological studies indicate that individuals with epilepsy are more likely to experience various types of headaches, particularly migraines, compared to those without epilepsy. Numerous studies emphasize the importance of both differential diagnosis and comorbidity due to shared symptoms between certain headache types and epilepsy, as reflected in current headache classifications. In this context, the relatively good attitudes and knowledge levels among headache patients are also promising in terms of public awareness [15, 16, 17, 18].

Although multiple sclerosis patients, who constituted the third largest group in our study (6.3%), had a low risk of developing epilepsy, their attitude scores remained within the acceptable limits as shown in Figure 1. Although rare, seizures are an important clinical feature of inflammatory demyelinating disorders of the central nervous system [19].

In our study, the smallest diagnostic group (0.6%), consisting of patients with Behçet’s disease patients, demonstrated insufficient knowledge, but they were the group with the best results on the attitude scale, as shown in Figures 1 and 2. Epileptic seizures are rarely observed in Behçet’s disease patients [20]. Although not very significant due to the small sample size representing this diagnosis, it can be considered positive data regarding the stigma problems surrounding epileptic patients. We also assessed stroke patients, who are at the highest risk for developing comorbid epilepsy regarding their knowledge and attitudes, as shown in Figure 1 and Figure 2. Stroke is a significant risk factor for the development of epilepsy. It has been shown that epilepsy may develop in 3–13% of all stroke cases in one third of the cases occurring in the elderly population [21]. The level of knowledge and attitude was low in 19 patients (5.7%) in our study. Since the criterion of not having had a seizure was sought in the stroke patients included in the study, the stroke group was limited to a very small group of 5.7% of all diagnoses. Therefore, it may not be objective enough to attribute this result to all stroke patients, but it can still be taken into consideration, as it constitutes a warning result that awareness is not sufficient.

Epilepsy awareness, in terms of the level of knowledge and attitude, is essential for society as a whole; however, for individuals at risk of seizures, having accurate information and a positive attitude is of even greater significance and can be vital to their safety and well-being. Our study is the first in the literature to examine the knowledge and attitude levels of individuals who may have higher empathy levels for epilepsy than the general population, even if they do not have a diagnosis of epilepsy or a history of seizures.

Both our study and existing literature in the field indicate that epilepsy awareness, adequate information, and the adoption of appropriate attitudes should be broadly promoted within societies, and that substantial progress is still required in this area. According to a study designed as a reference for researchers closely examining this subject and monitoring its progression, the knowledge of the Hungarian population about epilepsy and their attitudes toward individuals with the condition have been systematically studied and documented since 1994. According to the results of this study, some parameters of familiarity and attitude indicators towards epilepsy have improved, but it was emphasized that they still need to be supported by educational programs and campaigns [22].

Several limitations were identified upon the completion of our study. Within the predefined study period, the number of eligible individuals who met the inclusion criteria and consented to complete the entire questionnaire remained limited to 331. The number of participants could not be increased due to their lack of interest in the study topic or their unwillingness to devote time to it. Consequently, the sample size could not be further expanded. Moreover, the representation of certain diagnostic subgroups was notably low, resulting in a lack of homogeneity across diagnostic categories. Although the study was conducted in a high-volume tertiary neurology outpatient clinic and offers valuable insight into epilepsy awareness among this specific patient population, the findings are not generalizable to the broader Turkish population due to potential cultural and regional variations.

## 5. Conclusion

By selecting with neurological conditions other than epilepsy, we were able to assess a randomly selected subgroup of adults without epilepsy. As in many similar studies, attitudes and knowledge about epilepsy were reviewed along with various accompanying parameters to gain insight into epilepsy awareness. The results of this and similar studies will help increase public awareness of epilepsy, empower individuals with epilepsy to approach their illness more consciously, reduce sociocultural challenges caused by both self and social stigma, and enable these individuals to integrate more effectively and effectively into society.

## Figures and Tables

**Table 1:** Participants’ responses on the epilepsy knowledge scale.

Questions of knowledge scale	Correct answern (%)
1. Epilepsy has many different types. (T)	97 (29.3)
2. Most people with epilepsy can work. (T)	231 (69.8)
3. Most children with epilepsy can go to public schools. (T)	261 (78.9)
4. Patients with epilepsy can be dangerous to others during a seizure. (F)	246 (74.3)
5. Some seizures may last for a matter of seconds. (T)	132 (39.9)
6. For most patients with epilepsy, seizures can be controlled with drugs. (T)	215 (65)
7. Brain surgery can be used to treat epilepsy in some cases. (T)	156 (47.1)
8. Most people with epilepsy have normal intelligence. (T)	232 (70.1)
9. Patients with epilepsy can be as successful at work as others. (T)	212 (64)
10. An epileptic seizure is caused by abnormal function of the nerve cells in the brain. (T)	239 (72.2)
11. Epilepsy is a kind of incurable disorder. (F)	173 (52.3)
12. Inadequate sleep, stress, and taking alcohol can cause a seizure. (T)	260 (78.5)
13. When you see a person having a seizure, you can stop the seizure by giving him/her an onion to smell. (F)	196 (59.2)
14. Patients with epilepsy can lead normal lives. (T)	213 (64.4)
15. Some kinds of seizures can be hardly noticed by others. (T)	82 (24.8)
16. When you see a person having a seizure, you should spill water on his/her face to stop the seizure. (F)	237 (71.6)

2 T: True, F: False.

**Table 2:** Participants’ responses on the epilepsy attitude scale.

Questions of attitude scale	1 point n (%)	2 point n (%)	3 point n (%)	4 point n (%)	5 point n (%)	Mean
1. If I had epilepsy, I would hide it from my friends	5 (1.5)	40 (12.1)	16 (4.8)	101 (30.5)	169 (51.1)	4.17
2. I would stay away from a friend if I know she/he had epilepsy.	0 (0)	5 (1.5)	13 (3.9)	81 (24.5)	232 (70.1)	4.63
3. I would date someone who has epilepsy (X)	26 (7.9)	34 (10.3)	127 (38.4)	99 (29.9)	45 (13.6)	3.31
4. I would object to hiring someone who has epilepsy	2 (0.6)	2 (0.6)	23 (6.9)	88 (26.6)	216 (65.3)	4.55
5. I would be embarrassed if someone in my family had epilepsy	0 (0)	11 (3.3)	11 (3.3)	68 (20.5)	241 (72.8)	4.62
6. I would object to the marriage of my child with someone who has epilepsy	3 (0.9)	25 (7.6)	114 (34.4)	74 (22.4)	115 (34.7)	3.82
7. I would marry someone who has epilepsy (X)	15 (4.5)	37 (11.2)	163 (49.2)	72 (21.8)	44 (13.3)	3.28
8. I would not trust a doctor with epilepsy, if I knew of his/her illness	0 (0.0)	11 (3.3)	16 (4.8)	69 (20.8)	235 (71)	4.59
9. I prefer to stay away from someone with epilepsy	1 (0.3)	8 (2.4)	10 (3)	51 (15.4)	261 (78.9)	4.70
10. Having epilepsy is something to be embarrassed about	1 (0.3)	3 (0.9)	12 (3.6)	38 (11.5)	277 (83.7)	4.77
11. I feel uncomfortable working with someone who has epilepsy	0 (0)	2 (0.6)	16 (4.8)	39 (11.8)	274 (82.8)	4.76
12. I feel comfortable with someone who has epilepsy (X)	23 (6.9)	8 (2.4)	52 (15.7)	96 (29)	152 (45.9)	4.04
13. I think patients with epilepsy are frightening	1 (0.3)	2 (0.6)	16 (4.8)	56 (16.9)	256 (77.3)	4.70
14. I think people with epilepsy are not physically attractive	1 (0.3)	3 (0.9)	23 (6.9)	42 (12.7)	262 (79.2)	4.69

2 X: Favorable attitude, all except X: Unfavorable attitude

**Table 3:** General characteristics of the participants.

Categories	
**Diagnosis**	**n (%)**
Headache	102 (30.8)
Cervical radiculopathy	50 (15.1)
Vertigo	46 (13.9)
Multiple sclerosis	21 (6.3)
Carpal tunnel syndrome	20 (6)
Stroke	19 (5.7)
Sleep disorder	16 (4.8)
Essential tremor	14 (4.2)
Polyneuropathy	12 (3.6)
Neuropathic pain	11 (3.3)
Parkinson’s disease	10 (3)
Syncope	8 (2.4)
Behçet’s disease	2 (0.6)
**Sex**	
Female	170 (51.4)
Male	161 (48.6)
**Age group**	
<40 age	176 (53.2)
≥40 age	155 (46.8)
**Marital status**	
Single	119 (36)
Married	181 (54.7)
Widow	10 (3)
Divorced	21 (6.3)
**Highest education status**	
Primary school	7 (2.1)
Secondary School	12 (3.6)
High school	152 (45.9)
University	142 (42.9)
Postgraduate	18 (5.4)
**Education level**	
Low education	171 (51.7)
High education	160 (48.3)
**Occupational status**	
Working	202 (61)
Not working	111 (33.5)
Student	18 (5.4)

**Table 4:** Participants’ responses regarding epilepsy.

Questions	n (%)
**Have you ever seen an epileptic seizure?**	
Yes	160 (48.3)
No	171 (51.7)
**Have you heard anything about epilepsy before?**	
Yes	248 (74.9)
Internet	91 (36.6)
Environment	157 (63.4)
No	83 (25.1)
**Have you ever read anything about epilepsy?**	
Yes	131 (39.6)
Internet	108 (82.4)
Book	23 (17.6)
No	200 (60.4)
**Do you know anyone with epilepsy?**	
Yes	179 (54.1)
Friend	68 (38)
Relative	39 (21.8)
Family	16 (8.9)
Neighbour	31 (17.3)
Another patient in hospital	25 (14)
No	152 (45.9)

**Table 5:** Comparison of the knowledge scale total score according to categorical parameters.

Parameters	Knowledge scale total score	p*
**Age groups**		
<40 age	9.44 ± 3.84 10 (0–16)	0.377
≥40 age	9.88 ± 4.03 10 (1–25)	
**Education level**		
Low education	8.54 ± 3.97 9 (0–16)	**<0.001**
High education	10.84 ± 3.53 11.5 (0–25)	
**Based on knowing someone with epilepsy**		
Who do not know anyone with epilepsy	8.23 ± 4 9 (0–16)	**<0.001**
Who know someone with epilepsy	10.89 ± 3.42 11 (2–25)	
**Sex**		
Female	9.78 ± 3.82 10 (1–16)	0.638
Male	9.51 ± 4.05 10 (0–25)	

Mean ± SD and median (min–max)

* Mann Whitney U test

**Table 6:** Comparison of the attitude scale total score according to categorical parameters.

Parameters	Attitude scale total score	p*
**Age groups**		
<40 age	60.95 ± 7.9 62.5 (6–70)	0.053
≥40 age	60.08 ± 6.66 62 (37–70)	
**Education level**		
Low education	59.29 ± 8.91 62 (6–70)	**0.046**
High education	61.89 ± 4.86 62 (41–70)	
**Based on knowing someone with epilepsy**		
Who do not know anyone with epilepsy	59.51 ± 6.74 61 (35–70)	**<0.001**
Who know someone with epilepsy	61.45 ± 7.74 63 (6–70)	
**Sex**		
Female	60.17 ± 8.44 62 (6–70)	0.947
Male	60.94 ± 5.98 62 (35–70)	

Mean ± SD and median (min–max)

* Mann Whitney U test
